# Later‐Stage Diagnosis and Poorer Survival in Pancreatic Cancer Patients With Vague Symptoms: A Population‐Based Study

**DOI:** 10.1002/ueg2.70231

**Published:** 2026-05-15

**Authors:** Vinay Murali Prasad, Helen G. Coleman, Sinead T. Hawkins, Helen Mitchell, Stephen McCain, Dorothy Johnston, Damien Bennett

**Affiliations:** ^1^ School of Medicine, Dentistry and Biomedical Sciences Queen's University Belfast Northern Ireland UK; ^2^ Patrick G Johnston Centre for Cancer Research Queen's University Belfast Northern Ireland UK; ^3^ Northern Ireland Cancer Registry Centre for Public Health Queen's University Belfast Northern Ireland UK; ^4^ Belfast Health and Social Care Trust Belfast Northern Ireland UK

**Keywords:** alarm, emergency, pancreatic cancer, referral, stage, survival, symptoms, vague

## Abstract

**Background:**

Most pancreatic cancer (PC) patients present symptomatically to cancer services, are diagnosed at late stage, and have poor survival. Patients with alarm symptoms (AS), such as jaundice or altered stool/urine colour, may be more readily recognised within current referral pathways than those with vague symptoms (VS).

**Objective:**

To examine the associations of presenting symptom category (alarm vs. vague) with route to diagnosis, stage at diagnosis, treatment, and survival in PC patients.

**Methods:**

Of 540 primary PC cases identified in the population‐based Northern Ireland Cancer Registry PC Audit (2019–2020), 28 were excluded because referral pathway information was insufficient to classify route to diagnosis, leaving 512 patients for analysis. Patients were categorised by presenting symptoms as AS or VS. *χ* ^2^ tests assessed group differences; Kaplan‐Meier and Log‐Rank tests compared 1‐year survival. Cox proportional hazards models compared mortality risk, adjusting for sex, age, socioeconomic status, referral route, tumour location, and stage at diagnosis. All statistical tests were two‐sided, and *p* < 0.05 was considered statistically significant.

**Results:**

More patients presented with VS (60.4%) than AS (39.6%). Emergency admission was more common in AS than in VS patients (66.0% vs. 33.7%, *p* < 0.001). VS patients more often had stage IV disease (62.1% vs. 39.9%, *p* < 0.001) and were less likely to receive tumour‐reductive treatment (34.0% vs. 50.2%, *p* < 0.001). One‐year survival was poorer in VS than in AS patients (24.9% vs. 33.7%, *p* = 0.001). VS was associated with a 48% increased mortality risk [HR = 1.48 (1.14–1.91)] after full adjustment.

**Conclusion:**

PC patients with VS were more often presented via GP; they had later‐stage disease and poorer survival. VS remained independently associated with poorer survival. Greater awareness of PC symptoms, especially VS, together with effective diagnostic pathways for non‐specific symptoms, may help support more timely investigation.

## Key Summary

- Summarise the established knowledge on this subject:
  ◦ Pancreatic cancer (PC) is usually diagnosed at late‐stage and has poor survival, with little improvement over recent decades.
  ◦ Presenting symptoms may be associated with the diagnostic route, stage at diagnosis, and access to treatment.
  ◦ Alarm symptoms (AS), such as jaundice or altered urine/stool colour, may be more likely to trigger urgent investigation and earlier diagnosis than vague symptoms (VS), such as abdominal pain or weight loss.
  ◦ The impact of VS presentation on treatment and survival in patients with PC has not been studied in population‐level datasets.
- What are the significant and/or new findings of this study?
  ◦ In PC patients, VS were more common than AS (60.4% vs. 39.6%) and were associated with later‐stage diagnosis (Stage IV: 62.1% vs. 39.9%) and lower likelihood of receiving tumour‐reductive treatment (34.0% vs. 50.2%).
  ◦ AS patients more often presented via emergency departments (66.0% vs. 33.7%), whereas VS patients more frequently presented via GP referral (39.2% vs. 23.2%), and were less often diagnosed within 31 days of referral.
  ◦ One‐year survival was significantly worse in VS than in AS patients (24.9% vs. 33.7%). On fully adjusted analysis, including adjustment for stage at diagnosis, having VS was an independent predictor of poorer survival, with an almost 50% increased hazard ratio [HR = 1.48 (1.14–1.91)].
  ◦ Our findings support the need for improved symptom awareness among the public and healthcare staff, and for diagnostic pathways (e.g., rapid diagnostic centres) that better accommodate patients, especially those with vague symptoms.

## Graphical abstract

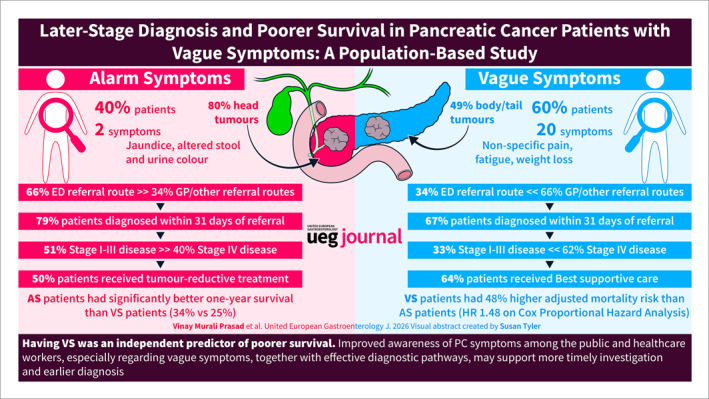

## 1 Introduction

Pancreatic cancer (PC) (ICD‐O‐3: C25) is the 10th most common cancer in the UK (2016–18), and the 5^th^ leading cause of cancer death, representing a major public health challenge [1]. The Northern Ireland (NI) Pancreatic Cancer Audit of patients diagnosed in 2019‐20, the first UK region to audit PC in recent years, provided a detailed insight into secondary care diagnostic and treatment pathways, focusing on population‐level outcomes [2]. The audit found that most PC patients had late‐stage disease and poor survival outcomes, consistent with previous studies [2, 3, 4].

Most PC patients present with symptoms before diagnosis, with 94% reporting symptoms when referred to NI cancer services in 2019–2020 [2]. PC symptoms have been categorised as alarm symptoms (AS) or vague symptoms (VS) in previous studies [5, 6]. AS comprises jaundice, altered urine or stool colour (pale stools and dark urine), which may be more readily recognised within referral pathways because of their clearer association with pancreatic cancer [7, 8]. In contrast, VS include abdominal pain, unexplained weight loss, loss of appetite and early satiety, which are not as closely associated with PC and can be linked to other cancers and medical conditions [9, 10]. A previous study estimated that weight loss could be present for over 2 years before formal PC diagnosis, highlighting potential diagnostic delays in VS patients [7].

A cancer patient's pathway and outcomes are influenced by their referral route into cancer services and the time from symptom onset to diagnosis and treatment [5, 11, 12, 13]. PC patients often present to emergency departments (ED) or their General Practitioner (GP) and are subsequently referred to Hepato‐pancreato‐biliary (HPB) cancer services for diagnosis, staging, workup and treatment [11]. According to the 2015 UK National Institute for Health and Care Excellence (NICE) Guidelines, patients aged 40 and over with jaundice should follow a suspected cancer pathway [14, 15], while patients aged 60 and over with VS require a red‐flag referral and CT scan within two weeks [14, 16]. Although local Northern Ireland Cancer Network (NICaN) guidance includes PC within its broader upper GI cancer guidelines, it lacks PC‐specific referral guidelines. More research on vague and acute symptom presentation is needed to develop symptom‐based decision‐support tools to assist GPs' assessment and support more timely investigation [17].

This novel, population‐based study examines the associations of PC patients' presenting symptoms (AS or VS) with their route to diagnosis (RTD), stage at diagnosis, the treatment they receive and outcomes, including survival.

## 2 Materials and Methods

**FIGURE 1:**
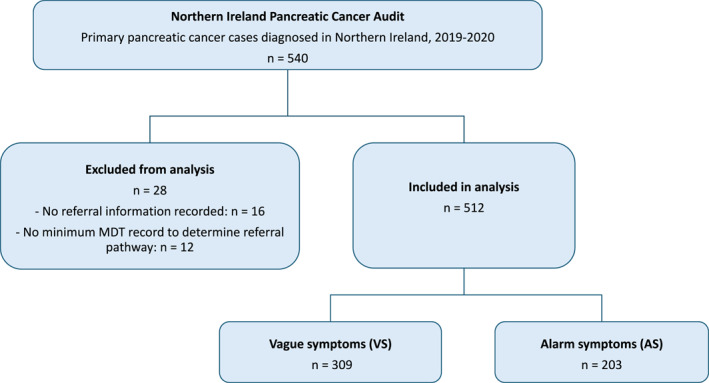
Study flow diagram for inclusion of pancreatic cancer patients in the analysis.

The NI Pancreatic Cancer Audit, a population‐based dataset of 540 primary PC cases diagnosed in NI in 2019–2020, was used in this study. In total, 28 cases were excluded because available audit data were insufficient to classify referral pathway/route to diagnosis. Of these, 16 had no recorded information on how patients were referred into the healthcare system, while a further 12 had no multidisciplinary team (MDT) meeting record available on the administrative pathway system. This does not necessarily mean that an MDT meeting did not occur; rather, the administrative process of recording the meeting had not taken place, leaving insufficient information to determine how patients entered secondary care, including whether referral was via GP or emergency presentation. The remaining 512 patients were included in the analysis (Figure 1). Patients were grouped into AS and VS categories (Supporting Information S1: Table S1) based on symptoms at initial presentation and in line with previous symptom classifications [5, 6]. AS included jaundice and altered stool or urine colour, while VS encompassed 20 other symptoms such as weight loss, fatigue, and joint pain (Supporting Information S1: Table S1).

*χ* ^2^ tests were used to assess differences in PC patient, pathway and treatment characteristics by symptom category (AS vs. VS), including:

- Sex—biological sex at birth (male/female)
- Age group—age at PC diagnosis (< 60 years/60–79 years/80+ years)
- Referral route—GP, emergency admission or other. “Other” included outpatient‐to‐outpatient referrals from another specialty, incidental findings, admission for another medical condition, and a residual “other” group. This residual group comprised cases recorded in the MDT administrative pathway system simply as “other”, for which no further breakdown could be obtained, together with referral sources that were not reported separately because of small numbers.
- Stage at diagnosis—based on UICC TNM classification, 8th edition [18].
- Tumour location (Head of pancreas/body and tail of pancreas/other location not otherwise specified)
- Treatment intent—curative, palliative anti‐cancer (PAC) or best‐supportive‐care (BSC). PAC includes tumour‐reducing interventions given with palliative intent. BSC indicates care without tumour‐directed treatment.
- Treatment delivery (tumour‐reductive)—surgery, chemotherapy and/or radiotherapy.
- Wait times from referral to diagnosis (≤ 31 days or > 31 days), and from referral to treatment (≤ 62 days or more than 62 days) as per UK Waiting Time targets [11].

Survival differences between AS and VS groups were assessed with Kaplan‐Meier (KM) curves and log‐rank tests. Using Cox proportional hazards (CPH) analysis, we compared the mortality risk in AS versus VS patients using unadjusted, partially adjusted (for sex and age at diagnosis) and fully adjusted hazard ratios (adding tumour location, socioeconomic status, referral types and stage at diagnosis). Socioeconomic status was defined by Northern Ireland Multiple Deprivation Measure 2017 (NIMDM2017) quintiles [19]. Analysis was conducted using Stata version 16. Adjusted survival curves were derived from the fully adjusted Cox proportional hazards model using Stata post‐estimation commands. All statistical tests were two‐sided, and a *p*‐value < 0.05 was considered statistically significant.

## 3 Results

Of the 512 PC patients, there were 274 males (53.5%) and 238 females (46.5%), with 85.7% aged over 60 years (Table 1). Fewer patients presented with acute (*n* = 203, 39.6%) compared with vague symptoms (*n* = 309, 60.4%). There were no significant differences in the proportion of patients presenting with acute and vague symptoms by sex, age group or deprivation quintile (Table 1).

**TABLE 1:** Patient characteristics by a symptom category.

Category	Sub‐category	Alarm Symptoms (%) *n* = 203	Vague symptoms (%) *n* = 309	*p*‐ value	Total (%) *n* = 512
Sex	Male	111 (54.7%)	163 (52.75%)	*p* = 0.669	274 (53.5%)
	Female	92 (45.3%)	146 (47.3%)		238 (46.5%)
Age group (Years)	< 60 yrs	27 (13.3%)	46 (14.9%)^a^	*p* = 0.711	73 (14.3%)
	60–79 yrs	123 (60.6%)	176 (57.0%)^a^		299 (58.4%)
	80+ yrs	53 (26.1%)	87 (28.2%)^a^		140 (27.3%)
Socioeconomic deprivation	Most deprived quintile 1	36 (17.7%)	61 (19.7%)	*p* = 0.823	97 (19.0%)
	Quintile 2	35 (17.2%)	46 (14.9%)		81 (15.8%)
	Quintile 3	45 (22.2%)	77 (24.9%)		122 (23.8%)
	Quintile 4	40 (19.7%)	62 (20.1%)		102 (19.9%)
	Least deprived quintile 5	47 (23.2%)	63 (20.4%)		110 (21.5%)
Referral	GP referral	47 (23.2%)	121 (39.2%)	P *≤* 0.001	168 (32.8%)
	Other	22 (10.8%)	84 (27.2%)		106 (20.7%)
	Emergency referral	134 (66.0%)	104 (33.7%)		238 (46.5%)
Stage at diagnosis	Stage I	43 (21.2%)	39 (12.6%)	*p* *≤* 0.001	82 (16.0%)
	Stage II	33 (16.3%)	27 (8.7%)		60 (11.7%)
	Stage III	28 (13.8%)	37 (12.0%)		65 (12.7%)
	Stage IV	81 (39.9%)	192 (62.1%)		273 (53.3%)
	Stage unknown	18 (8.9%)	14 (4.5%)		32 (6.3%)
Location of tumour	Head	162 (79.8%)	88 (28.5%)	*p* *≤* 0.001	250 (48.8%)
	Body + tail	22 (10.8%)	150 (48.5%)		172 (33.6%)
	Other NOS^b^	19 (9.4%)	71 (23.0%)		90 (17.6%)
Treatment intent	Curative	55 (27.1%)	48 (15.5%)	*p* = 0.001	103 (20.1%)
	Palliative anti‐cancer	50 (24.6%)	62 (20.1%)		112 (21.9%)
	Best supportive care	98 (48.3%)	199 (64.4%)		297 (58.0%)
Received tumour‐reductive treatment	Treatment received	102 (50.2%)	105 (34.0%)	*p* *≤* 0.001	207 (40.4%)
	Best supportive care	101 (49.8%)	204 (66.0%)		305 (59.6%)
Treatment type	Surgery	43 (21.2%)	43 (13.9%)	*p* = 0.031	86 (16.8%)
	No surgery	160 (78.8%)	266 (86.1%)		426 (83.2%)
	Chemotherapy	93 (45.8%)	79 (25.6%)	*p* *≤* 0.001	172 (33.6%)
	No chemotherapy	110 (54.2%)	230 (74.4%)		340 (66.4%)
	Radiotherapy	19 (9.4%)	11 (3.6%)	*p* = 0.006	30 (5.9%)
	No radiotherapy	184 (90.6%)	298 (96.4%)		482 (94.1%)

^a^ Rounding Error.

^b^ Not Otherwise Specified.

Emergency admission was the most common referral route (46.5%), followed by GP (32.8%) and other pathways (20.7%). Within the “other” referral category (*n* = 106), the largest subgroup was outpatient‐to‐outpatient referral from another specialty (38.7%), followed by admission for another medical condition (13.2%) and incidental findings (12.3%). The remaining 35.8% comprised a residual “other” group that could not be further disaggregated for reporting. A higher proportion of AS patients were referred following emergency admission compared to VS patients (66.0% vs. 33.7%, *p* < 0.001), with a lower proportion referred via GP (23.2% vs. 39.2%, *p* < 0.001). Overall, a majority of PC patients were diagnosed with late‐stage disease (53.3% stage IV vs. 40.4% Stage I‐III) (Table 1). However, fewer AS patients were diagnosed with late‐stage disease compared with VS patients (39.9% vs. 62.1%, *p* < 0.001) (Table 1). Tumours were most often anatomically located in the head of the pancreas (*n* = 250, 48.8%) than in the body or tail (*n* = 172, 33.6%) (Table 1), with a much higher proportion of AS patients having tumours in the head of pancreas compared to VS patients (79.8% (AS) versus. 28.5% (VS), *p* < 0.001), and conversely, a lower proportion having tumours in the body/tail (10.8% (AS) versus. 48.5% (VS), *p* < 0.001) (Table 1).

Most patients received best‐supportive‐care (BSC) (58.0%) as initial treatment intent, with fewer AS patients initially receiving BSC compared to VS patients (48.3% vs. 64.4%, *p* = 0.001), and a higher proportion receiving curative intent (27.1% vs. 15.5%, *p* = 0.001) (Table 1). Thus, more patients with AS received tumour‐reductive treatment compared with VS patients (50.2% vs. 34.0%, *p* < 0.001) (Table 1). Among those receiving tumour‐reductive treatment, more AS than VS had surgery (21.2% vs. 13.9%, *p* = 0.031), chemotherapy (45.8% vs. 25.6%, *p* < 0.001), and radiotherapy (9.4% vs. 3.6%, *p* = 0.006) (Table 1).

A higher proportion of AS patients were diagnosed within 31 days of referral compared to VS patients (78.8% vs. 67.3%, *p* = 0.005) (Table 2). Although a greater proportion of AS patients were treated within 62 days of referral, this difference was not significant (56.5% vs. 43.5%, *p* = 0.62) (Table 2).

**TABLE 2:** Wait times from referral to diagnosis and referral to treatment analysed by a symptom category.

Subgroup	Total (*n* = 512)	Time referral to diagnosis		*p* value	Total (*n* = 207)	Time referral to treatment		*p* value
		≤ 31 days	> 31 days			≤ 62 days	> 62 days	
Alarm symptoms	203 (39.6%)	160 (78.8%)	43 (21.18%)	0.005	102 (49.3%)	52 (56.5%)	50 (43.5%)	*p* = 0.62
Vague symptoms	309 (60.4%)	208 (67.3%)	101 (32.69%)		105 (50.7%)	40 (43.5%)	65 (56.5%)	

### 3.1 Survival

**FIGURE 2:**
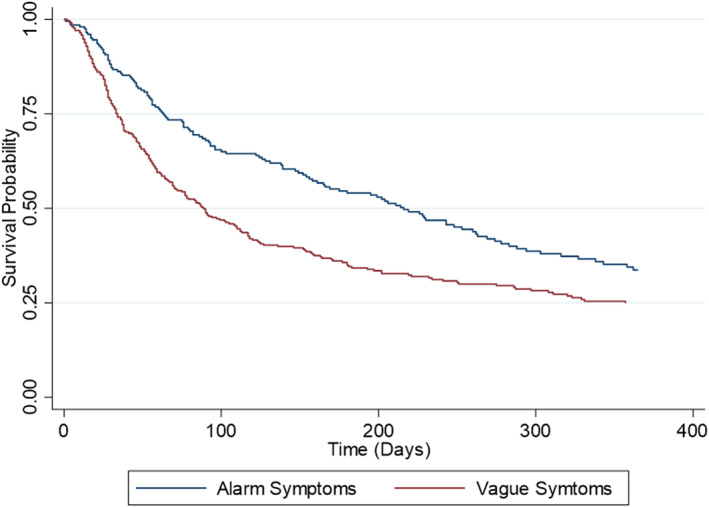
Kaplan–Meier curve showing One‐year survival differences between pancreatic cancer patients diagnosed 2019–2020 presenting with Alarm (AS) and Vague (VS) symptoms.

**FIGURE 3:**
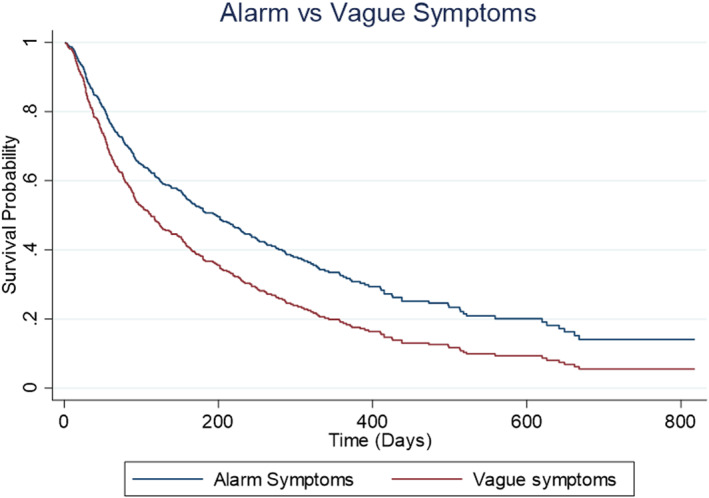
Adjusted survival curves for Pancreatic Cancer Patients diagnosed 2019‐2020 by Symptom Category. Curves were derived from the fully adjusted Cox proportional hazards model in Stata, adjusting for sex, age at diagnosis, socioeconomic status, referral route, tumour location and stage at diagnosis.

AS patients had significantly better 1‐year survival (33.7%) than VS patients (24.9%) (*p* < 0.001) (Figure 2, Table 3). In CPH analysis (Table 4), VS patients had higher mortality risk throughout: unadjusted HR = 1.42 (1.15–1.75), partially adjusted (sex, age) HR = 1.48 (1.20–1.83), and fully adjusted (socioeconomic status, referral route, tumour location and stage at diagnosis) HR = 1.48 (1.14–1.91). Adjusted survival curves derived from the fully adjusted Cox model are shown in Figure 3.

**TABLE 3:** KM 1‐year survival by a symptom category.

	Survival (days)	Survivor function	95% CI	Log rank
Alarm symptoms (*n* = 203)	90	68.0%	61.1%–73.9%	*p* = 0.001
	180	54.1%	46.9%–60.7%	
	365	33.7%	26.8%–40.8%	
Vague symptoms (*n* = 309)	90	48.9%	43.2%–54.3%	
	180	35.0%	29.6%–40.4%	
	365	24.9%	20.0%–30.2%	

Abbreviation: CI, Confidence Interval.

**TABLE 4:** Cox Proportional Hazards Ratio results for unadjusted, partially adjusted and fully adjusted models.

Symptom type	Deaths	Alive	Unadjusted HR (95% CI)	Partially adjusted HR (95% CI)^a^	Fully adjusted HR (95% CI)^b^
Alarm symptoms	140	63	1.00	1.00	1.00
Vague symptoms	236	73	1.42 (1.15–1.75)	1.48 (1.20–1.83)	1.48 (1.14–1.91)

Abbreviation: CI, Confidence Interval.

^a^ Partially adjusted includes sex and age at diagnosis.

^b^ Fully adjusted includes sex, age at diagnosis, tumour location (head, body and tail, other), Stage at diagnosis, socioeconomic status, referral types (GP, emergency, other).

## 4 Discussion

Pancreatic cancer has one of the poorest cancer survival rates, with limited improvement over recent decades, and more than half of PC patients are still diagnosed with late‐stage metastatic disease [2, 4, 20]. A better understanding of PC patients' presenting symptoms may help clarify associations with stage at diagnosis, treatment options, and survival [4]. To the authors' knowledge, this is the first population‐based study which examines key characteristics of PC patients with acute and vague symptoms and investigates differences in RTD, stage at diagnosis, treatment received and survival outcomes. While some studies have suggested the categorisation of symptoms for PC patients, the relationship between symptom categories, clinical pathways and patient outcomes has not been examined in clinical settings [7, 21, 22]. Our finding that nearly 40% of PC patients had acute, and about 60% vague symptoms, provides valuable population‐level information on presenting symptoms prior to PC diagnosis [8]. More AS patients were diagnosed following emergency admission, while more VS patients were diagnosed following GP referrals, in line with the findings of Pearson et al., who investigated non‐specific symptoms in cancer patients presenting to primary care [5]. AS onset may prompt a more rapid healthcare‐seeking from patients and urgent escalation from healthcare staff, which could contribute to earlier investigation in some patients.

AS patients were more likely to have head‐of‐pancreas tumours, which can cause biliary obstruction and symptoms of jaundice and stool/urine discolouration, whereas VS patients were more likely to have body/tail tumours [23]. Our finding that AS patients were more likely to be diagnosed following ED attendance, rather than primary care, suggests these symptoms may be of particular concern for patients and healthcare staff. In contrast, patients with VS may present via GP with less specific symptoms that are more difficult to recognise as pancreatic cancer than the more obvious obstructive presentation seen in AS patients. A retrospective population‐based study in France linked longer treatment delay in PC patients mainly to the absence of jaundice [24]. Because data on the time from first symptom onset or first healthcare presentation to treatment were not available, we could not determine whether these differences reflected patient‐related delay, delay after presentation to healthcare services or both [25]. Previous studies have reported gender differences in healthcare interactions, with males often having fewer primary care appointments [26]. However, there was no significant difference in alarm/vague symptom profile between male and female PC patients (Table 1) and the significantly higher HR for VS compared to AS patients remained following adjustment for sex and age (Table 4).

Current NI guidelines recommend an urgent (red‐flag) referral for suspected cancer to HPB cancer services for patients presenting to their GP with jaundice, with associated waiting‐time targets [17]. In contrast, patients presenting with diarrhoea, back pain, abdominal pain, nausea, vomiting, constipation or new‐onset diabetes may require broader diagnostic consideration and more investigations before referral [27]. Development of PC‐specific referral guidelines for GPs in the UK may support more timely investigation in patients with VS, and our study's findings on AS and VS patients could support protocolised HPB referrals [5]. A previous UK study suggested that diagnostic delay may be due to difficulties in both primary and secondary care referrals rather than public delays in health‐seeking behaviours [28]. However, because we did not have data on symptom onset, first healthcare contact or the number of primary care consultations, we could not distinguish patient‐related delay from delay occurring after presentation to healthcare services. A qualitative study of PC patients found that vague, non‐specific, intermittent symptoms preceding a PC diagnosis can often be dismissed as unimportant, with one patient saying, “it can't be very important because it comes and goes” (Supporting Information S1: Table S1) [29]. Back pain, dyspepsia, and fatigue are often attributed to benign conditions, delaying help‐seeking and diagnosis [10, 29, 30]. Their fluctuating nature may give false reassurance to patients and clinicians alike, leading to later presentation and reduced likelihood of curative treatment. As with Pearson's study, we found that a lower proportion of PC patients with VS were diagnosed within 31 days of initial referral compared to those with AS (67% vs. 79%) [5]. Support for assessment and interpretation of VS in primary care may help facilitate a more timely investigation of PC.

We found significant differences in tumour stage at diagnosis between AS and VS patients, with a much higher proportion of VS patients having late‐stage disease (62.1%) compared to AS patients (39.9%). This may reflect differences in symptom recognisability, tumour location, and diagnostic pathway complexity [7]. However, our data do not allow us to determine whether this pattern is driven primarily by patient help‐seeking behaviour, post‐presentation diagnostic processes or both. Although epigastric pain is recognised as an important red flag in surgical contexts, we classified abdominal pain as a vague symptom due to diagnostic ambiguity in primary care, where it often overlaps with benign conditions and is rarely recorded with anatomical precision. In our dataset, symptom documentation did not reliably distinguish between epigastric and non‐epigastric pain, limiting our ability to stratify symptom location. Furthermore, pain related to PC is known to be heterogeneous and may present as poorly localised or diffuse abdominal discomfort, particularly in early stages [31]. Our approach aligns with previous literature highlighting the challenges of symptom interpretation in GP consultations, where most PC symptoms have low specificity and are documented with variability [9]. While unintentional weight loss is a red flag in suspected cancer diagnoses, especially in primary care guidelines, its diagnostic value varies depending on the context. In PC specifically, weight loss is a common presenting symptom—seen in around 70% of patients prior to diagnosis, including early‐stage cases [32]. However, evidence also suggests that it may be under‐recognised or misattributed by patients or clinicians, potentially contributing to delayed presentation or diagnosis [33]. Weight loss often reflects systemic cachexia linked to advanced PC, reducing resectability and survival. Though not inherently “vague,” it may be overlooked early in PC—reinforcing the complexity of using it as a trigger for timely investigation [34]. Our results are similar to those of Pearson et al., who found that more VS patients were diagnosed with later‐stage disease (32%) compared to AS patients (21%), although their study was not specific to PC [5]. A previous study found tumour stage to be an independent predictor of survival as part of a multivariate model in PC patients [35]. However, in our study, we found that VS remained an independent predictor of poorer survival even after adjusting for stage at diagnosis.

Patients with VS had significantly poorer 1‐year survival than those with AS, highlighting the need for diagnostic pathways that better accommodate non‐specific symptom presentations [5]. Stage is likely to account for an important part of this survival difference, with a higher proportion of VS patients having stage IV disease and a lower proportion receiving tumour‐reductive treatment. However, in fully adjusted Cox pH analysis, which accounted for stage at diagnosis, VS patients still had almost 50% higher mortality risk compared to AS patients (HR 1.47). A Danish primary care based study of colorectal cancer patients found that those first presenting with VS had higher 3‐year mortality and longer pathways compared with those with AS [36]. More broadly, patients diagnosed via GP referral generally have better outcomes than those diagnosed through ED, although symptom profiles are often not considered in such comparisons. Consistent with this, the Routes to Diagnosis (RTD) project in England found that patients diagnosed via emergency presentation had significantly poorer 1‐year survival compared to those diagnosed through GP referral [37]. Indeed, Pancreatic Cancer UK reported 1‐year survival was nearly three times better following GP referral compared with emergency referral for PC patients, but again, the symptom profile was not considered [20]. In our cohort, however, the poorer outcomes observed in VS patients despite a higher proportion being diagnosed via GP referral, likely reflect the more diagnostically challenging nature of vague symptom presentation rather than a less favourable effect of the referral route itself.

Governments, healthcare providers and third sector stakeholders have developed policies to support earlier cancer diagnosis and shifts to an earlier stage disease to improve patient outcomes [38, 39, 40]. Efforts to reduce the proportion of patients presenting with late‐stage disease may be supported by better use of symptom appraisal tools, such as the UK‐based QCancer risk tool, by healthcare staff, including GPs, and by improving symptom awareness among the public as recommended in the NHS Long Term Plan [7, 39]. Symptom appraisal tools may help GPs better interpret patient presentation patterns and reduce variability in patient outcomes while ensuring cost‐effective use of resources and preventing over‐investigation [9, 21, 41, 42, 43]. Rapid diagnostic centres (RDCs) have recently been introduced in NI and may allow timelier cancer diagnosis via GP referral, especially for VS patients [5, 44]. RDCs provide a diagnostic pathway for patients with non‐specific symptoms, such as unexplained weight loss, abdominal pain and recent onset of fatigue, that could be associated with cancer, by reducing multiple referrals and improving the speed and efficiency of diagnostic pathways [45]. Public‐facing symptom awareness initiatives may also play an important role, particularly for persistent or evolving non‐specific symptoms that might otherwise be normalised or overlooked. Such initiatives may support earlier help‐seeking [20], although we did not have patient data on symptom appraisal or help‐seeking behaviour. However, the effectiveness and efficiency of RDCs will still need to be assessed compared to cancer cases diagnosed via GPs or EDs [46, 47].

To the authors' knowledge, there are no recent studies linking PC symptoms with the pattern of treatments offered to patients. During GP interactions, factors such as stage and tumour location are unknown, making the investigation of factors such as symptom profile, gender, age and socioeconomic status on treatment patterns offered to patients more important. Our study suggests that VS presentation is associated with lower receipt of tumour‐reductive treatment and highlights the need for further education and research regarding non‐specific symptom presentations [29]. Measures to improve diagnostic pathways for VS patients may support more timely investigation, reduce emergency admissions, and expand treatment options. However, diagnosing pancreatic cancer swiftly via general practice is challenging [48], with PC vague symptoms often overlapping with other medical conditions, making timely, cost‐effective triage and referral difficult.

Strengths of this study include using a national, population‐based cohort of all patients diagnosed with PC in NI during 2019–2020, which, unlike other studies, makes it generalisable to other populations. The dataset used high‐quality electronic health records from the NICR, which are broadly representative of UK population‐based health datasets. Limitations include a lack of information on the severity or duration of symptoms, as primary care data were not available. In particular, the dataset did not include symptom onset, symptom severity, health‐seeking behaviour or the interval from first symptom to first healthcare contact; therefore, patient‐related delay could not be distinguished from diagnostic or treatment intervals occurring after referral. As we only had access to secondary care data, the number of GP visits made by the patient and the tests and investigations performed in primary care prior to diagnosis could not be determined. Linkage to GP datasets to examine these questions would be a useful extension of this study, especially regarding the development of new‐onset primary diabetes or worsening glycaemic control, which can be early risk factors for PC [49].

A further consideration is that the study period included 2020, during the COVID‐19 pandemic. We were unable to extend the analysis to additional years because symptom‐level data were collected as part of the NI Pancreatic Cancer Audit, which covered the years 2019–2020 and are not available in routine NICR data collection. However, the impact of the COVID‐19 pandemic on incidence, stage at diagnosis and short‐term survival across all cancer types has been examined by NICR, which found that for pancreatic cancer patients in NI there was no significant difference in case distribution by stage, hospital admission type or treatment delivery within six months of diagnosis in 2020 compared with 2018–2019, supporting inclusion of 2020 and 2019 data in the present study [50].

## 5 Conclusion

The majority of PC patients present symptomatically, are diagnosed with late‐stage disease, and have poor survival, with limited improvement in these trends in recent decades. Our study has shown that PC patients with AS had earlier‐stage disease, more emergency presentations, more tumours in the head of the pancreas and more treatment options than VS patients. In contrast, patients with VS had more advanced disease at diagnosis, were more likely to present to GPs than emergency EDs, had more pancreatic body/tail tumours and received less tumour‐reductive treatment. On fully adjusted analysis, including adjustment for stage at diagnosis, VS remained associated with poorer survival, with an almost 50% increased hazard ratio. Better symptom awareness among the public and healthcare staff and better use of effective symptom appraisal tools by healthcare staff, alongside ongoing RDC pilots, may support more timely investigation and earlier diagnosis for PC patients, especially those with vague symptoms.

**Abbreviations**
AS: Alarm Symptoms
BSC: Best Supportive Care
HPB: Hepato‐pancreato‐biliary
HSC: Health and Social Care
KM: Kaplan Meier
NI: Northern Ireland
NICR: Northern Ireland Cancer Registry
PAC: Palliative Anti‐Cancer
PC: Pancreatic Cancer
RTD: Routes to Diagnosis
VS: Vague Symptoms

## Funding

This work was supported by funding from Northern Ireland Pancreatic Cancer (NIPANC) for the Northern Ireland (NI) Pancreatic audit. NIPANC is a local NI charity which is focused on raising awareness, supporting individuals affected by pancreatic cancer, and funding research in Northern Ireland. The funders played no role in the study design, data collection or analysis, decision to publish or preparation of the manuscript. The Northern Ireland Cancer Registry (NICR) is funded by the Public Health Agency of NI. This uses data collected by health services as part of their care and support functions.

## Ethics Statement

NICR has ethical approval from the Office for Research Ethics Committees of Northern Ireland (ORECNI) (Ref: 20/NI/0132, IRAS project ID: 288121), for the collection and use of routinely collected data relating to cancer patients within the fields of health and social care research. As such, specific ethical approval and informed consent from the data subjects were not required for this study. Data processing and analysis was conducted in accordance with the NICR Confidentiality and Data Protection Policies in accordance with the ORECNI conditions for ethical approval to use these data.

## Conflicts of Interest

The authors declare no conflicts of interest.

## Supporting information


**Table S1:** List of Alarm symptoms and list of Vague symptoms recorded in NI Pancreatic Cancer Audit 2019–2020.

## Data Availability

The datasets generated and/or analysed during the current study are not publicly available due to the limits of the ethical approval granted to the NICR to share patient level data. Anonymised, non‐patient level data can be made available on reasonable request.
