# Supplementary material for: Later‐Stage Diagnosis and Poorer Survival in Pancreatic Cancer Patients With Vague Symptoms: A Population‐Based Study
Source: United European Gastroenterol J. 2026 May 15;14(4):e70231. doi: 10.1002/ueg2.70231 (PMC13177201; doi:10.1002/ueg2.70231)
Supplement: Supplementary file 1 — Table S1: List of Alarm symptoms and list of Vague symptoms recorded in NI Pancreatic Cancer Audit 2019–2020. [file UEG2-14-e70231-s001.docx]

**Supplementary Tables**

**Supplementary Table 1**. List of Alarm symptoms and list of Vague symptoms recorded in NI Pancreatic Cancer Audit 2019-2020

| Alarm symptoms (2) | Vague symptoms (20) |
| --- | --- |
| - Jaundice - Altered stool / urine colour | - Abdominal pain - Abdominal swelling - Fatigue - Joint Pain - Low mood - Pale - Duodenal obstruction - Weight loss - Nausea and vomiting - Decreased appetite - Back pain - Diarrhoea - Indigestion - Palpable abdominal mass - Itching - Blood clot - Pain on eating - Palpable liver - Cachexia - Other symptoms - Asymptomatic |
